# Viral Induced Microbial Mortality in Arctic Hypersaline Spring Sediments

**DOI:** 10.3389/fmicb.2016.02158

**Published:** 2017-01-23

**Authors:** Jesse Colangelo-Lillis, Boswell A. Wing, Isabelle Raymond-Bouchard, Lyle G. Whyte

**Affiliations:** ^1^Department of Earth and Planetary Science, McGill University, MontrealQC, Canada; ^2^McGill Space Institute, McGill University, MontrealQC, Canada; ^3^Department of Natural Resource Science, McGill University, MontrealQC, Canada

**Keywords:** viral dynamics, hypersaline sediments, polar microbial ecology, oligotrophy, lysogeny

## Abstract

Viruses are a primary influence on microbial mortality in the global ocean. The impacts of viruses on their microbial hosts in low-energy environments are poorly explored and are the focus of this study. To investigate the role of viruses in mediating mortality in low-energy environments where contacts between viruses and microbes are infrequent, we conducted a set of *in situ* time series incubations in the outlet and channel sediments of two cold, hypersaline springs of the Canadian High Arctic. We found microbial and viral populations in dynamic equilibrium, indicating approximately equal birth and death rates for each population. *In situ* rates of microbial growth were low (0.5–50 × 10^3^ cells cm^-3^ h^-1^) as were rates of viral decay (0.09–170 × 10^4^ virions cm^-3^ h^-1^). A large fraction of the springs’ viral communities (49–100%) were refractory to decay over the timescales of our experiments. Microcosms amended with lactate or acetate exhibited increased microbial growth rates (up to three-fold) indicating organic carbon as one limiting resource for the microbial communities in these environments. A substantial fraction (15–71%) of the microbial populations contained inducible proviruses that were released- occasionally in multiple pulses- over the eight monitored days following chemical induction. Our findings indicate that viruses in low-energy systems maintain low rates of production and activity, have a small but notable impact on microbial mortality (8–29% attenuation of growth) and that successful viral replication may primarily proceed by non-lethal strategies. In cold, low biomass marine systems of similar character (e.g., subsurface sediments), viruses may be a relatively minor driver of community mortality compared to less energy-limited environments such as the marine water column or surface sediments.

## Introduction

Sunlight and photosynthesis are the drivers for surface life and environments sufficiently connected to utilize macromolecules synthesized in the photic zone (e.g., marine surface sediments). In environments decoupled from photoautotrophy, the source of electron potential can limit microbial growth. Examples of these environments include aquifers, subsurface lakes, glacial beds, permafrost environments, marine subsurface sediments, and any surface environment whose chemistry inhibits the growth of photosynthetic life (e.g., Don Juan pond; Siegel et al., 1979). Environments lacking also in a substantial contribution of chemoautotrophic carbon fixation can be thought of as ‘low energy’ habitats, characterized by low biomass, low rates of microbial growth, and low rates of metabolism (Hoehler and Jørgensen, 2013). Microbes in these environments exhibit adaptation to low nutrient fluxes (e.g., amino acid catabolism; Zinser and Kolter, 1999) and their investigation provides an opportunity to explore ecological processes of habitats that make up a substantial fraction of the contemporary biosphere (e.g., subsurface marine sediments; Fry et al., 2008). This work focuses on viral predation on microbes under low energy conditions.

Viral dynamics, including rates of production and decay, combined with rates of microbial growth, inform a variety of processes in microbial ecology. Since the recognition of vast numbers of viruses in the ocean, research has focused on the impact of these entities on their microbial hosts (Bergh et al., 1989; Fuhrman, 1999). Initial dynamics research correlated TEM enumeration of virus-like particles (VLPs) with changes in microbial abundances across space and time, demonstrating environmental viral activity and primarily infection of microbes (Bratbak et al., 1990, 1996; Hennes and Simon, 1995). Recognition of the extent of microbial infection and lysis as a control on microbial abundance, maintaining populations far below resource-controlled levels was a milestone in microbial oceanography (Lenski, 1988; Proctor and Fuhrman, 1992). Following characterization of community-scale rates of viral lysis came an appreciation of the role of viruses in both maintaining microbial diversity and in shunting organic carbon from a particulate to a dissolved state, thereby maintaining that carbon in oceanic surface waters (Proctor and Fuhrman, 1990; Waterbury and Valois, 1993; Hennes et al., 1995; Middelboe et al., 1996; Gobler et al., 1997). Investigations addressing whether high viral abundances might be maintained by resistance to decay found that viruses in sea water are quick to decay, implying that their production is similarly rapid in the ocean and that interactions between microbes and viruses are dynamic on short time scales (Suttle and Chen, 1992; Wilhelm et al., 1998).

As an alternative to lytic replication, a significant fraction of viruses are capable of lysogenic replication, wherein the viral genome is integrated into that of its host, replicating along with host until an induction event signals the virus to excise from its host genome and revert to lysis (Burnett, 1934). The frequency of lysogens (microbes with at least one viral genome integrated into their own) in the aqueous marine environment varies widely between environments and seasons (Paul, 2008). Significantly, despite a high frequency of lysogens in the environment, natural induction of lysogens is rare, and the vast majority (97% or more) of viruses observed in sea water are the result of lytic infections (Wilcox and Fuhrman, 1994). Provirus (a viral genome integrated into its host’s) genes are known to occasionally be expressed, altering the phenotype of their microbial host in a process termed lysogenic conversion (Barksdale and Arden, 1974). Genomics research indicates viruses are responsible for a substantial amount of genetic exchange in marine microbial communities (McDaniel et al., 2010). In tandem, viruses simultaneously significantly impact both community scale processes (mortality and diversity) as well as genetic composition and gene flow within the microbial community (Rohwer and Thurber, 2009).

A longstanding hypothesis proposes that viral dynamics are controlled by trophic state, as assessed by the rate of primary productivity and the abundances of biologically essential nutrients (Weinbauer et al., 1993). The rationale behind this hypothesis is that eutrophic environments support higher concentrations of microbes (and consequently viruses) than oligotrophic systems, and that viruses will have greater impact on their hosts at higher concentrations. This hypothesis has been addressed across trophic gradients in aqueous environments (Corinaldesi et al., 2003; Bongiorni et al., 2005; Cheng et al., 2010). By extension, this hypothesis suggests there may be conditions under which microbial growth and abundance are sufficiently low such that lytic viral replication, which depends on host activity, is effectively inhibited. However, in every environment studied to date, viruses have been found to exert some influence on microbial mortality.

The low energy deep subsurface habitats on Earth are inaccessible to *in situ* experimental manipulation. As a proxy towards understanding what role viruses may play in microbial mortality there, this work focused on more easily accessible shallow subsurface environments, exhibiting reduced biomass, rates of metabolism and growth compared to surface environments. Two perennial cold hypersaline springs on Axel Heiberg Island in the Canadian High Arctic were investigated. These springs have a number of physical and chemical differences between their near-anoxic, cold outlets and progressively warmer and more oxygenated channels and serve as a natural extension of the conditions in which the trophic control hypothesis has been addressed. Further, compared to marine water and surface sediments, these systems are characterized by low viral abundances, virus to microbe ratios, and virus-microbe contact rates, suggesting that viruses in these springs play a lesser role in controlling microbial populations through lytic activity than in the marine environment, but a comparable role to the role they play in subsurface sediments (Colangelo-Lillis et al., 2016b). Here we quantitatively evaluate this suggestion with *in situ* time series experiments to monitor microbial growth, viral production and provirus induction from lysogens. Processes that differ in comparing these springs to surface environments may point toward the nature of the same processes in even more energy limited environments (e.g., deep subsurface marine sediments), characterized by even lower biomass and metabolic rates.

## Materials and Methods

### Site Description

All samples were collected from spring sediments in the proximity of the McGill Arctic Research Station on Axel Heiberg Island in July of 2015 (Supplementary Figure S1; Pollard et al., 2009). Prior to surface exposure, spring water ascends through > 400 m of Carboniferous anhydrite evaporites and permafrost (Jackson and Harrison, 2006), thereby setting the Na-, Ca-, Cl-, SO_4_-rich geochemistry of the springs. We studied two springs (Gypsum Hill and Lost Hammer) that have exhibited stable geochemistry over the last 10 years (Supplementary Table S1).

**FIGURE 1:**
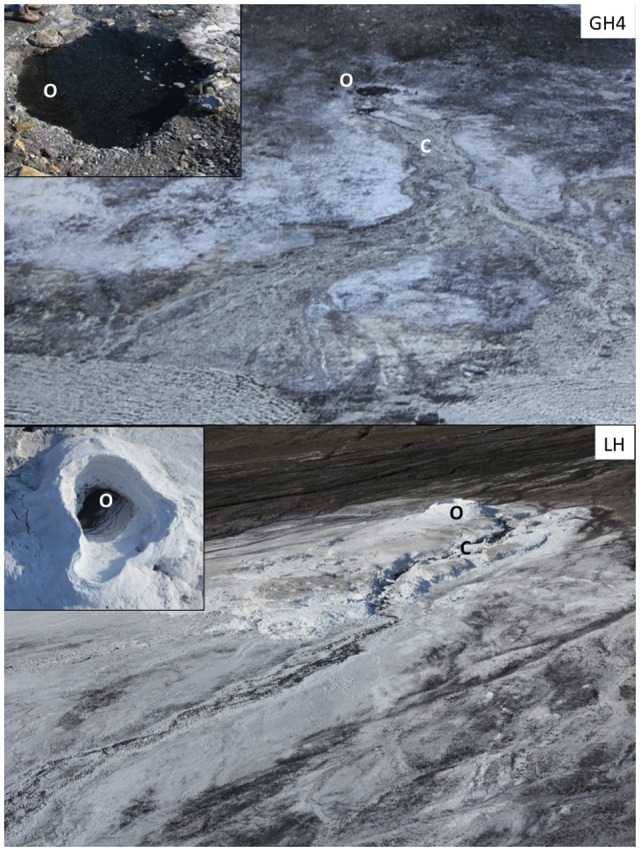
**Sampling sites.** Hypersaline Arctic springs Gypsum Hill 4 (GH4) and Lost Hammer (LH) investigated for viral dynamics. Outlet (O) and channel (C) experimental stations are indicated for each spring. Photos of each outlet as viewed from above are inset; GH4 outlet pool diameter is 2 m, LH outlet pool diameter is 3 m.

Gypsum Hill Springs emerge from the southern base of Gypsum Hill via ≈40 outlets that flow over ≈30 m of permafrost, in shallow, dispersive channels terminating into Expedition River (**Figure 1**). The outlet of the largest spring (GH4) discharges at a rate of ≈1 L s^-1^, forming a 1 m deep, 2 m wide, circular pool with roiling sediments as a result of water and gas discharge. Over the past decade (Supplementary Table S1), outlet waters have been perennially uniform at 4.2–6.9°C, pH 7.4–7.7, 75–100‰ salinity, microoxic (2.5–6.3 μM dissolved oxygen) and reducing (Eh between -318 and -97 mV). These channels contain mixed sediments (fine to coarse sand and pebbles) that are variably coated with a veneer of travertine precipitate (Omelon et al., 2006). Major spring water ions include chloride, sodium, sulfate, and calcium (Pollard et al., 1999). Microbial and molecular characterization has identified Bacterial and Archaeal genes associated with aerobic and anaerobic heterotrophic and autotrophic metabolisms, including sulfur- and sulfate- reducing bacteria, methanogens, sulfur oxidizing bacteria and methanotrophs (Perreault et al., 2007, 2008; Niederberger et al., 2009).

Lost Hammer Spring (LH) emerges from a single outlet and has formed a 3 m high halite tufa (**Figure 1**). In summer months, water flows through the base of this structure; in winter months the hydrohalite structure freezes and outlet water overflows the tufa lip. Outlet waters are perennially uniform (Supplementary Table S1) at ≈-5°C, pH ≈6.5, 240‰ salinity, and are microoxic (0.6 μM dissolved oxygen) and reducing (Eh -187 mV). The abundant ions of LH are the same as GH4, in similar ratios but greater concentrations (Niederberger et al., 2010). Microbial and molecular characterization from both the spring outlet and channel indicate a low diversity community and the presence of anaerobic methanotrophs, methanogens, sulfur oxidizing bacteria and ammonia oxidizers (Niederberger et al., 2010; Lay et al., 2012, 2013; Lamarche-Gagnon et al., 2015). The biomass of both springs are very low, with the channel sediments of each inhabited by an order of magnitude more microbes than the outlet of the same spring (Colangelo-Lillis et al., 2016b). Collectively the absence of chlorophyll, low biomass and low rates of metabolism suggest the outlets of these springs to be low energy environments (Perreault et al., 2007; Colangelo-Lillis et al., 2016b).

### Sediment Measurements

Pore water measurements included temperature and concentrations of dissolved oxygen, nitrate, nitrite, ammonia, sulfide, sulfate, and dissolved and solid phase organic carbon. Dissolved oxygen was measured at 3 cm below sediment surface using a Picollo2 fiber-optic oxygen meter (Pyroscience, Aachen, Germany). Pore water was collected into 15 mL syringes through Rhizon (Rhizosphere, Wageningen, Netherlands) tubing inserted 3 cm into the sediment, and measured for aqueous chemistry immediately upon recovery using CHEMetrics kits (Midland, Virginia; K6923, K7003, K1503, K9523, K9203) and a portable spectrometer (V-2000, CHEMetrics). Sediment porosity was calculated from sediment density and mass reduction following drying at 100°C for 24 h. Total organic carbon and total nitrogen content were measured on an Elemental Analyzer (NC 2500, CE Instruments, Wigan, UK), following acid removal of inorganic carbonates as carbon dioxide at the GEOTOP stable isotope laboratory at UQAM (Montreal, QC, Canada). Dissolved organic carbon was measured on a Total Organic Carbon Analyzer (TOC-V CHS; Shimadzu, Tokyo) at the GEOTOP Environmental Organic Geochemistry laboratory at Concordia University (Montreal, QC, Canada).

### Microcosms

Microcosms for all dynamics experiments were assembled on site in July 2015. Microcosm experiments were conducted in 6 mL serum vials. All vials were filled with surface (0–5 cm depth) sediment (80% volume) and spring water (20% volume), and immediately capped with butyl rubber stoppers underwater. Microcosm experiments were established at four sampling stations (**Figure 1**). Incubations were performed *in situ* in sediments of GH4 in both the spring outlet and channel (5 m downstream of the spring outlet) sediments. LH microcosms were taken from the spring site and incubated in a permafrost freezer at the McGill Arctic Research Station at -3 ± 1°C. Following incubation, microbial processes were terminated by the addition of formaldehyde (final concentration 2%) using sterile syringes. Formaldehyde and all other treatment reagents were 0.02 μm-filtered prior to use. Fixed samples were maintained at 4°C through transport and storage until enumeration.

### Microbial Growth

Microbial growth and viral production microcosms employed non-native nucleotide 5-ethynyl-2′-deoxyuridine (EdU; Invitrogen A10044). This synthetic nucleotide was incorporated into newly replicated nucleic acids, which could later be identified using click chemistry between the nucleotide and a fluorescent azide (Alexafluor488-azide, Invitrogen A10266; Ferullo et al., 2009; Qu et al., 2011; Cieślar-Pobuda and Los, 2013; Wang et al., 2013). At each spring site, 60 μL of 5 mM EdU (final concentration 50 μM) was injected into nine 6 mL microcosms using sterile syringes. Two additional vials served as no treatment controls to normalize for any effects of EdU addition or bottling effects-wherein *in situ* conditions and processes are influenced by the artificial replication of their environment (Pernthaler and Amann, 2005). Single EdU-treated vials were formaldehyde fixed at time points: 0, 1, 2, 4, 8, 12, 24, 48, and 96 h. Untreated vials were fixed at time points 0 and 96 h. At each spring site, eight additional 6 mL microcosms were assembled and EdU treated as above. Four of these vials were amended with lactate and another four with acetate (60 μL of 1 M carbon source, final concentration 10 mM). Per spring site, EdU ± carbon treated vials were formaldehyde fixed at time points: 0, 8, 24, and 96 h. Lactate was chosen because it can be utilized by many sulfate-reducers, known to be present and active in these sediments (Perreault et al., 2007; Lay et al., 2013). Acetate was chosen as it is among the most simple and ubiquitously utilized carbon sources, and has been employed in culturing isolates from LH (Niederberger et al., 2010).

Microbial growth rates (μ) were calculated both from the average change in microbial abundance at each point in a time series (μ_Δcells_, cells cm^-3^ h^-1^), and separately from the average change in percentage of new cells having incorporated EdU (μ_Δ%EdU-cells_, % new cells cm^-3^ h^-1^) at each point in a time series. As EdU incorporation is less than 100% efficient, its efficiency was calculated as the ratio of the growth rate determined from changes in % cells with incorporated EdU to the growth rate determined from changes in cell numbers. This efficiency factor (calculated for carbon amended microcosms) facilitated conversion from % cells with incorporated EdU to *in situ* growth rate in non-carbon amended microcosms, where the growth and death rates were equal and no changes in absolute cell numbers could be observed. Average microbial turnover rates across the time series were estimated by dividing μ by microbial abundance. Theoretically EdU incorporation should be detectable in viruses generated from microbes that had incorporated the nucleotide; however, high background fluorescence resulting from click-chemistry reactions on Anodisc 0.02 μm filters made assessment of viral production rates infeasible using this method.

### Viral Decay

Endogenous viral production was terminated by addition of the bacteriostatic KCN, and decay was measured as the disappearance over time of directly countable viral particles (Heldal and Bratbak, 1991). At each spring site, 60 μL of 0.2 M KCN pH 7.0 (final concentration 2 mM) was injected into ten 6 mL microcosms using sterile syringes; two additional vials per site served as no treatment controls to normalize for any immediate effects of KCN addition or bottling effects. Single KCN-treated microcosms were formaldehyde fixed at time points: 0, 1, 2, 4, 8, 12, 24, 48, 96, and 144 h. Untreated vials were fixed at time points 0 and 96 h. Decay rates were calculated from linear best fits to a plot of viral abundance with time.

### Provirus Induction

Provirus induction microcosms employed DNA-damaging agent mytomycin C to induce provirus excision from their host genomes, replication and lysis (Ackermann and DuBow, 1987). At each spring site, 0.1 mL of 60 μg mL^-1^ (final concentration 1 μg mL^-1^) was injected into six 6 mL microcosms using sterile syringes; two additional vials served as no treatment controls to normalize for any immediate effects of mytomycin C addition or bottling effects. Single mytomycin C-treated microcosms were formaldehyde fixed at time points: 0, 8, 24, 48, and 96 and 144 h. Untreated vials were fixed at time points 0 and 96 h. The fraction of lysogens was calculated as the reduction in microbial abundance associated with the induction divided by the starting population abundance. Temperate (capable of lysogenic replication) viral burst sizes were determined from the ratio of increase in VLP abundance (VLP cm^-3^) to the decrease in microbial abundance (cells cm^-3^) over the apparent duration of the induction event. When possible, the decay rate of the induced viral community was calculated as above.

### Sediment Extraction

Prior to filtration, 3.0 cm^3^ aliquots of sediments were washed 2X with 1X PBS to remove excess salt, then disrupted in extraction buffer (0.1% sodium pyrophosphate, 0.5% Tween20, in 1X PBS) first by agitation on a shaker (Scientific Instruments Vortex Genie 2, setting 7, 20 min) and then by sonication (Heat Systems Ultrasonics W185D, 100 W). This extracted sample was underlain with 2 mL 50% Histodenz (Sigma D2158; 1.3 g mL^-1^) and centrifuged at 500 *g* for 15 min at 4°C. Supernatant was retained for filtration and enumeration of microbes and viruses, and extracted sediments were dried overnight at 60°C and weighed to determine sediment density and porosity. All chemicals were 0.02 μm-filtered prior to use.

### Microbial and Viral Abundance

Our enumeration of microbial and viral abundances followed established techniques (Suttle and Fuhrman, 2010). Sediment extracts were filtered through 0.02 μm pore size Anodisc filters, stained with 40X SYBR Green I, and enumerated at 1000X magnification using a Nikon Eclipse 80i microscope and NIS-Elements BR imaging software (v3.2). Per sample, between 200 and 500 microbes and VLPs were counted from 1140 fields of 100 μm^2^ each. Given the microscope’s field of view at 1000X magnification, each sample was effectively ‘measured’ 15 times, leading to a relative uncertainty of each abundance measurement of <25% (defined as standard deviation of all measurements divided by the mean of all measurements). Visibly dividing cells (VDC) were also enumerated. Viral counts were back-corrected to *in situ* concentrations using the storage decay relationships described in Colangelo-Lillis et al. (2016b). Methods employed here did not distinguish between Bacteria, Archaea, and small single-celled Eukaryota. Members of each domain have been reported in both springs (Perreault et al., 2007, 2008; Niederberger et al., 2010; Lay et al., 2012, 2013) and are herein collectively referred to as microbes.

### Click-Chemistry Fluorescent Tagging of EdU

Microbes and viruses from samples incubated with EdU were prepared as described above for viral and microbial abundances. Parallel samples were prepared on 0.2 μm pore size, black nucleopore filters for determining percentage of cells with incorporated EdU. Samples prepared on nucelopore filters retained only microbes, and not VLPs. All washes, EdU-labeling and counter stain were performed in microcentrifuge tubes. Fixed microbes were pelleted from sediment extractions by centrifugation at 5000 × *g* at 4°C. The supernatant was decanted and the cells were resuspended in 1.5 mL 1X PBS and transferred to microcentrifuge tubes. After pelleting, the supernatant was again removed and the pellet was resuspended gently in 100 μL Triton X-100 (0.5% in PBS) and incubated at room temperature for 20 min. Following incubation, cells were centrifuged and washed once in 1.5 mL PBS. The cell pellet was resuspended in 250 μL PBS to which 250 μL 2X fluorophore-azide reaction mixture was added. Microbes were incubated at room temperature (protected from light) for 20 min and then collected by centrifugation. The pellet was washed with 1.5 mL PBS, resuspended in 250 μL PBS to which 250 μL Hoechst 33342 (4 μg mL^-1^) was added. Microbes were incubated again at room temperature (protected from light) for 15 min and then collected by centrifugation. The pellet was washed with 1.5 mL PBS and finally resuspended in 1.5 mL PBS and filtered on to a 0.2 μm pore size nucleopore filter for enumeration. Similar methods were employed on 0.02 μm pore size anodiscs to attempt a determination of viruses with incorporated EdU. However, background fluorescence prevented meaningful enumeration.

## Results

### Sediment Characteristics

Measurements of sediment physical and chemical characteristics were consistent with those made from the same springs over the past decade (**Table 1**; Supplementary Table S1). In both Gypsum Hill (GH4) and Lost Hammer (LH) spring outlets, sediment pH was circumneutral (7.0 and 6.8), temperature (4.2 and -3.6°C) and dissolved oxygen (≤1.3 and 0.6 μM) were low and oxidation reduction potential (ORP) values were negative (-287 and -181 mV). Measurements of each of these parameters yielded greater values in channel sediments compared to outlet sediments (**Table 1**). Solid phase organic carbon was 0.17 and 0.33 wt % in GH4 and LH outlets and was 2–3X greater in the channels of GH4 and LH. Solid phase nitrogen was 0.02 wt % in both springs’ outlets and increased 1.5–2X in channel sediments. Dissolved organic carbon was only measured from GH4; replicate measurements were 233 and 783 μM in outlet sediments and 308 and 508 μM in channel sediments.

**Table 1:** Sediment physical measurements and pore water chemistry.

Spring	Station	Distance from outlet (m)	Temp. (°C)	pH	ORP (mV)	Density (g cm^-3^)	Porosity (%)	Dissolved oxygen (μM)	Total carbon (wt%)	Total organic carbon (wt%)	Dissolved organic carbon (μM)	Total nitrogen (wt%)	Sulfate (mM)	Sulfide (μM)	Ammonium (mM)	Nitrate (μM)	Nitrite (μM)
GH4	Outlet	0	4.2	7.0	-287	3.02	48	0–1.3	0.74	0.17	233, 783	0.02	41	53	10.6	15	0.29
GH4	Channel	5	7.6	7.2	-330	3.51	54	38-110	0.72	0.44	308, 508	0.04	39	179	0.3	26	0.43
LH	Outlet	0	-3.6	6.8	-181	2.67	42	0–0.63	0.29	0.33	na	0.02	75	75	0.2	^∗^	^∗^
LH	Channel	5	-0.6	7.0	-120	2.82	49	34–69	0.76	0.50	na	0.03	75	<19	0.06	^∗^	^∗^

*^∗^Sum of nitrate and nitrite values reported in Lay et al. (2012) as 58 mM for LH outlet sediments and 1.8–2.4 mM for LH channel sediments.*

### *In situ* Microbial and Viral Abundances

**FIGURE 2:**
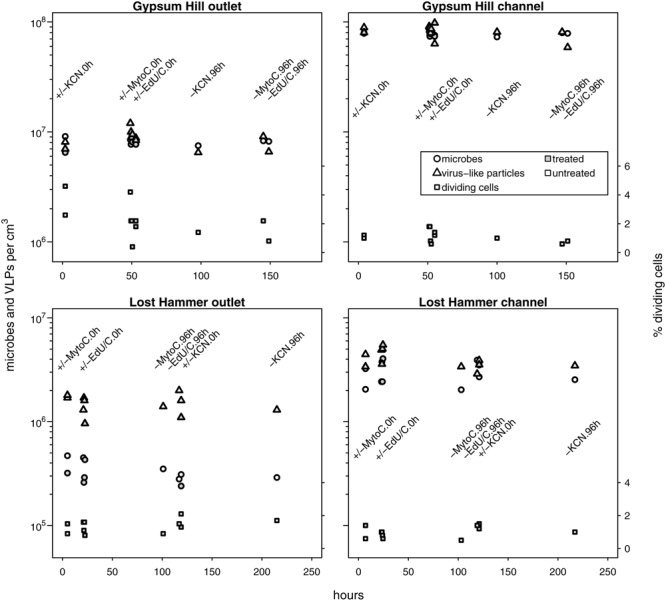
***In situ* microbial and VLP abundances collated from growth, decay and induction experiments.** Measured values from no-treatment controls (time-0 and -96 h) and time-0 h experimental treatment microcosms are shown. Microbial and VLP abundances are indicated by circles and triangles, respectively; the percentage of visibly diving cells is indicated by squares (right hand *y*-axis values). Open shapes indicate no treatment, shaded shapes indicate treatment (KCN for decay experiments, mytomycin C for induction experiments, EdU ± lactate or acetate for growth experiments). Experimental treatment and incubation times (0 or 96 h) are specified by text in line with column of measured values. Time 0 h for GH4 incubations corresponds to 1200 July 15, 2015; time 0 h for LH incubations corresponds to 1200 July 11, 2015. For each spring station, these data points were used to estimate uncertainties for microcosm time series samples without biological replicates. Note logarithmic left hand *y*-axis.

**FIGURE 3:**
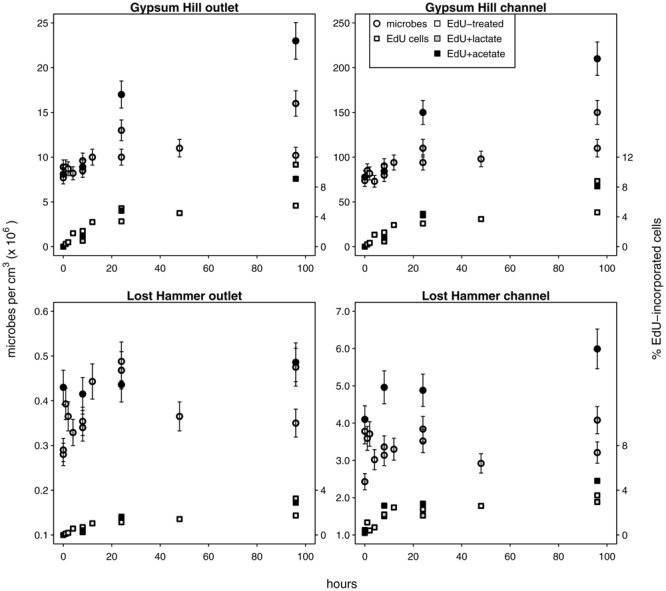
**Microbial growth in unamended and carbon amended microcosms.** Circles indicate total microbial abundances, the percentages of microbes with incorporated EdU are indicated by squares (right hand *y*-axis values). Unshaded shapes indicate EdU treated samples; shaded shapes indicate samples additionally treated with lactate (gray) or acetate (black). Note linear left hand *y*-axis.

**FIGURE 4:**
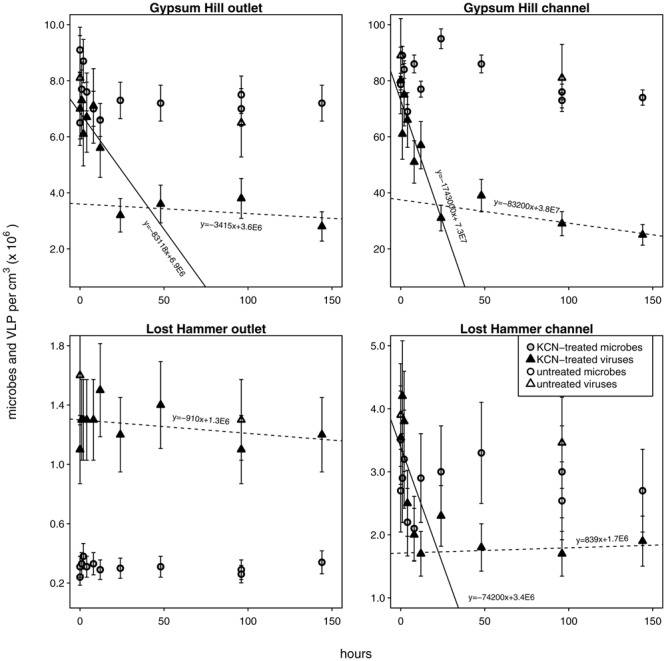
**Viral decay microcosms.** Circles indicate microbial abundances, VLPs are indicated by triangles. Unshaded shapes indicate untreated control samples; shaded shapes indicate samples treated with KCN. Gray and black shading are only for clarity in distinguishing VLP from microbial abundances. Solid ‘realized average’ lines indicate labile viral decay rates; dashed lines indicate refractory viral decay rates (realized average line equations are indicated). For each station the intersection of the lines indicates the abundance of viruses refractory to decay. Note linear left hand *y*-axis.

**FIGURE 5:**
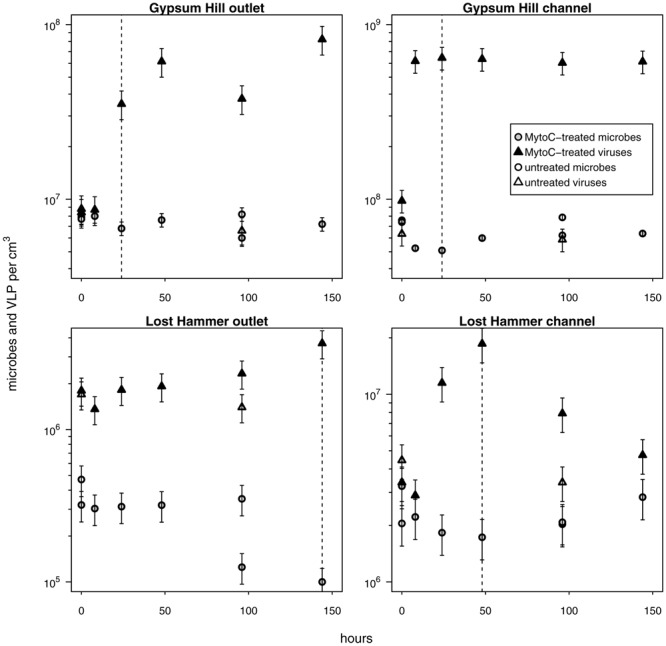
**Provirus induction microcosms.** Shapes and shading as in Figure 4; shaded shapes indicate values from mytomycin C treated samples. For each station, the duration of the induction period considered in calculations of percentage of lysogens and lysogen burst size is indicated by a vertical dashed line. Note logarithmic left hand y-axis.

Untreated samples fixed at time 0 and 96 h, and treated samples fixed at time 0 h for each experiment (i.e., growth, decay, and induction; 11 microcosms per spring station) yielded estimates for microbial and viral abundances that were consistent with single mean value and standard deviation at each station (**Figure 2**; **Table 2**). Microbial abundances were 8.0 ± 0.7 × 10^6^, and 7.7 ± 0.3 × 10^7^ cells cm^-3^ for GH4 outlet and channel, and 3.4 ± 0.8 × 10^5^ and 3.0 ± 0.8 × 10^6^ cells cm^-3^ for LH outlet and channel. Viral abundances were 8.6 ± 1.6 × 10^6^ and 8.2 ± 1.2 × 10^7^ VLP cm^-3^ for GH4 outlet and channel, and 1.5 ± 0.3 × 10^6^ and 4.0 ± 0.8 × 10^6^ VLP cm^-3^ for LH outlet and channel. We did not measure multiple experimental microcosms at intervening time points for each experiment. We therefore take the relative variability of these replicate microcosms as our best estimate of the uncertainties for abundance measurements for all treated samples for each experiment within a spring station (**Figures 3–5**). The percentage of VDC was between 0 and 5% for all samples (2.2 ± 1.3, 1.1 ± 0.4% for GH4 outlet and channel and 1.4 ± 0.4, and 1.0 ± 0.3% for LH outlet and channel). ‘Bottle effects’ refer to situations where the *in situ* conditions and processes are influenced by the artificial replication of their environment (Pernthaler and Amann, 2005). We tested for these situations by comparing both treated samples from the start of the experiments and untreated samples from the end of the experiment to untreated samples from the start of the experiment. Untreated samples, chemically fixed for enumeration at both time 0 and at 96 h for each experiment, within each spring station, contained comparable abundances of viruses and microbes to treated samples fixed at time 0 h. Thus no discernible ‘bottle effects’, resulting from either establishing the microcosms, or from adding experimental treatment chemicals were observed, and no corrections for these influences were made (**Figure 2**).

**Table 2:** Viral dynamics measurements.

Spring	Station	Microbes (cm^-3^)	VDC (%)	Viruses (cm^-3^)	VMR	Microbial growth (cm^-3^ h^-1^)	Microbial turnover (h)	Labile viral population (%)	Labile viral decay (cm^-3^ h^-1^)	Labile viral turnover (h)	Refractory viral population (%)	Refractory viral decay (cm^-3^ h^-1^)	Refractory viral turnover (h)	Burst size	Microbial mortality (%)	Inducible microbes (%)
GH	Outlet	8.0 × 10^6^	2.2	8.6 × 10^6^	1.1	5.0 × 10^4^	160	47	8.3 × 10^4^	49	53	3.4 × 10^3^	1335	21	8	15
GH	Channel	7.7 × 10^7^	1.1	8.2 × 10^7^	1.1	4.3 × 10^5^	180	51	1.7 × 10^6^	24	49	8.3 × 10^4^	483	22	18	34
LH	Outlet	3.4 × 10^5^	1.4	1.5 × 10^6^	4.4	5.4 × 10^2^	630	0	na	na	100	9.1 × 10^2^	1648	9	19	71
LH	Channel	3.0 × 10^6^	1.0	4.0 × 10^6^	1.3	2.1 × 10^4^	140	42	7.4 × 10^4^	23	58	0	∞	12	29	42

*VDC, visibly dividing cells; VMR, virus:microbe ratio.*

### Microbial Growth

We used C-amended microcosms to calculate the efficiency of EdU incorporation into new microbes and the intrinsic growth rates at each station. The efficiency of EdU incorporation into new cells in acetate-amended microcosms was 2–19%, and 6–27% in lactate-amended microcosms (Supplementary Table S2). Realized microbial growth rates (calculated between 0 h and each successive time series point) ranged from 5.4 × 10^2^ to 4.3 × 10^5^ microbes cm^-3^ h^-1^ (**Figure 3**; **Table 2**). In each spring, growth rates were higher in the channel sediments compared to the outlet, and growth rates in GH4 were greater than those in LH. Microbial growth rates in microcosms amended with organic carbon were greater than the intrinsic growth rates and varied from 5.6 × 10^2^ to 1.1 × 10^6^ microbes cm^-3^ h^-1^ (**Figure 3**; Supplementary Table S2). In GH4 sediments acetate increased growth rates more so than lactate (by an average factor of 2.5). In LH outlet sediments lactate increased growth rate more than acetate (factor of 2.7), and in LH channel sediments carbon addition apparently exerted a small inhibitory affect on growth (3–20%; Supplementary Table S2).

### Viral Decay

Viral decay rates varied from 9.1 × 10^2^ to 1.7 × 10^6^ viruses cm^-3^ h^-1^ (**Figure 4**; **Table 2**). In GH4 outlet and channel, and LH channel, decay was initially relatively rapid, then quickly subsided, leaving a refractory viral pool that decayed at a slower or unobservable rate (**Figure 4**; **Table 2**). The refractory viral population made up 49–58% of the initial populations of these spring stations. The entire viral population of LH outlet exhibited a decay rate comparable or lower than the refractory populations of other stations. Given evidence that the viral and microbial communities were in dynamic equilibrium, initial decay rates were taken as equivalent to *in situ* viral production rates, and following numerous reports (Heldal and Bratbak, 1991; Guixa-Boixereu et al., 1999; Fischer and Velimirov, 2002), the viral production rates that balanced the initial (labile) viral pool decay rates were employed in calculations of viral-induced microbial mortality.

### Provirus Induction and Fraction of Lysogens

In each induction time series, microbial abundance decreased and viral abundance increased following exposure to mytomycin C. The apparent time to respond to the inducing agent and duration of induction varied between experimental stations (**Figure 5**); these induction periods (24–144 h) were used to assess the fraction of lysogens in each station. Lysogens made up 15–71% of the microbial populations, with the largest proportion in the coldest, highest salinity, lowest biomass spring station (LH outlet; **Table 2**). Lysis of induced lysogens may have stimulated microbial growth (see **Figure 5**, channel stations time series), and our time series did not capture the full extent of the induction event in all spring stations (**Figure 5**, outlet stations time series). As a result, estimates of the fraction of microbes that are lysogens should be considered minimum values. Temperate virus burst sizes were similar between stations within each spring: 21–22 viruses cell^-1^ in GH4 and 9–12 viruses cell^-1^ in LH. The microbial response to induction was sufficiently rapid and uniform in channel sediments to allow for estimates of decay rates of induced temperate phage. Compared to *in situ* populations, induced temperate phages decayed more slowly in GH4 channel sediments (20% *in situ* rate), and more quickly in LH channel sediments (200% *in situ* rate; Supplementary Table S3).

## Discussion

This work describes viral dynamics experiments performed in cold, oligotropic sediments of polar hypersaline spring sediments in the Canadian High Arctic in order to address two questions: (1) Is there is a direct relationship between trophic conditions and the predatory influence exerted by viruses on their microbial hosts? (2) Are viruses capable of lysogenic replication more common in oligotrophic environments? These questions form the basis for the trophic hypothesis of viral influence on microbial growth (Corinaldesi et al., 2003) and have implications for microbial ecology and evolution. By nature of their low temperature, low carbon availability, and low microbe and viral abundances, the springs investigated in this study represent an experimentally accessible, low energy environment in which to test the hypotheses above.

### Microbial and Viral Populations Are in Dynamic Equilibrium

Constant *in situ* abundances of microbes and viruses and dividing cells are consistent with microbial and viral abundances measured from the same spring sediments in summer of 2013 (Colangelo-Lillis et al., 2016b). These measurements suggest that microbial and viral populations in these sediments are each in dynamic equilibrium, both throughout our sampling period (July 11–18, 2015) and potentially across annual seasons (July; 2013 and 2015). Further, this dynamic equilibrium indicates that endogenous rates of viral production and decay are balanced. The abundances of viruses measured (≈10^6^ to 10^8^ viruses cm^-3^; **Figure 2**) were approximately 100-fold lower than those generally found in marine surface sediments (i.e., 10^8^–10^10^ viruses cm^-3^; Danovaro et al., 2008a), indicating different controlling influences on the communities in the surface sediments of these polar springs.

### Slow Microbial Growth Is Limited by Organic Carbon

*In situ* microbial growth varied from 0.05 to 43.0 × 10^4^ microbes cm^-3^ h^-1^ (**Table 2**). Growth in GH4 channel sediments was one order of magnitude greater than in its outlet, and two orders of magnitude greater in LH channel sediments compared to its outlet. Notably, in each spring a greater fraction of cells were found to be dividing from outlet stations compared to channel stations (**Figure 2**), indicating longer durations of the visible phase of cell division in the colder, less oxygenated outlet sediments. These differences in abundances and duration of cell division may be related to the availability of organic carbon or nitrogen as these nutrients varied ≈twofold between sites within a given spring (**Table 1**) and as carbon amended experiments resulted in increased growth (**Figure 3**). These differences are unlikely to be related to viral predation pressures, which are greater in channels compared to outlets (see below), or to protist predation, as protists were not observed in any sediment samples. Nutrient limited trophic conditions influence viral production by changing the size, metabolism, and growth rate of host cells (Hewson et al., 2001; Corinaldesi et al., 2003), primarily by decreasing the overall energy available to support the microbial community. Microbial production in marine surface sediments leads to between 2 and 130 ng C cm^-3^ h^-1^ (converted from reported g^-1^ values, 50% porosity and sediment density 1.8 g cm^-3^; Danovaro et al., 2008b) from sediments spanning a wide range of water depths and trophic conditions. Using a representative cell radius of 0.25 μm (based on TEM images of cells from these sediments; Colangelo-Lillis et al., 2016b) and a cellular carbon content of 310 fg C μm^-3^ (Danovaro et al., 2008a), production rates from GH4 and LH sediments fell between 1 and 872 pg C cm^-3^ h^-1^, underlying the limited energy availability in these sediments relative to marine surface sediments. We note that taxa-selective growth stimulation by labile carbon amendment may have occurred and affected our analyses of *in situ* community dynamics.

### Low Rates of Viral Production and Decay Indicate Sluggish Viral Dynamics

In even the most prolific of spring station sediments (GH4 channel, 1.7 × 10^6^ cm^-3^ h^-1^), viral production was lower than that seen in marine surface sediments (viral production from the lowest 5% of dataset was 9.8 ± 3.9 × 10^6^ cm^-3^ h^-1^; Danovaro et al., 2008b) or deep sea sediments (5.7 ± 1.4 × 10^7^ cm^-3^ h^-1^; Corinaldesi et al., 2007). These low viral production rates of GH4 and LH are consistent with the observed low microbial growth rates. In contrast to both shallow and deep-sea surface sediment viruses, described as highly dynamic and an active component of sediment ecosystems, with production rates ranging from 0.1 to 5 × 10^8^ cm^-3^ h^-1^ (Hewson and Fuhrman, 2003; Mei and Danovaro, 2004; Danovaro et al., 2008b), the viruses of these cold hypersaline spring sediment environments appear to be active, but at a greatly reduced rate.

In each spring the viral decay profile was interpreted to represent two viral communities: the first appeared prone to decay (labile) and the second refractory (**Figure 4**). A similar trend of rapid viral decay, followed by much more gradual or negligible decay has been reported from both water column (Heldal and Bratbak, 1991; Guixa-Boixereu et al., 1999; Fischer and Velimirov, 2002) and sediments (Fischer et al., 2004). In each spring, a substantial proportion of viruses (49–100%) fell into the refractory category (**Table 2**). The viral community from the LH outlet station was subject to a single decay rate, but given this rate was lower than the refractory viral decay rates from other spring stations, the entire viral population was considered refractory. Measurements of viral decay in sea water have led to estimates of viral turnover times ranging between 1 h and a few days (Suttle and Chen, 1992; Fuhrman and Noble, 1995; Noble and Fuhrman, 1997). These decay rates were correlated with the biological richness of the water, with the most rapid decay occurring in coastal waters and the slowest decay in oligotrophic offshore waters (Fuhrman, 1999). Investigations of viral decay in sediments have reported both rapid and very slow rates, leading authors to alternatively propose that virus preservation in sediments is either very poor (Mei and Danovaro, 2004) or very good (Middelboe et al., 2011; Dell’Anno et al., 2015). Viruses from GH4 and LH spring sediments appear to be in the later category, with turnover rates of the labile pool on the order of 10s of hours, and weeks to months for the refractory pool. The mechanism of this decay-resistance is unknown but could be related to the high concentration of salt, low concentrations of extracellular proteolytic enzymes, clay-adsorption and stabilization of viral particles, or structural modifications to viral protein coats specifically adapted to persist in environments with low host abundance and encounter rates.

### Viruses Are Not the Dominant Cause of Microbial Mortality

In order to evaluate viral induced microbial mortality, a measure of the viral burst size of a lysing cell is required. This value is frequently determined by direct assessment of visibly infected cells, but no such cells have been detected from these sediments (Colangelo-Lillis et al., 2016b). Here, burst size was inferred from provirus induction experiments, by dividing the number of temperate virions released by the concomitant decreases in microbial abundances (**Figure 5**, **Table 2**; Weinbauer and Suttle, 1999). These burst sizes are expected minimums; increases in burst size would force estimates of viral induced microbial mortality downward. These estimates of burst sizes are comparable to those found in other cold, hypersaline and/or oligotrophic environments (e.g., 15–28, Weinbauer and Suttle, 1999; 6–324, median = 16, Wells, 2008; 6–35, mean = 22, Guixa-Boixareu et al., 1996), but much lower than virus burst sizes reported from eutrophic environments (e.g., 33–64, Weinbauer and Suttle, 1999). Increasing latent periods and decreasing burst sizes (even the apparent cessation of infection) are associated with suboptimal nutrient concentrations (Proctor et al., 1993; Moebus, 1996). Low nutrient concentration also correlates with small cell size, another factor observed to decrease burst size (Choi et al., 2010), and observed in GH4 and LH sediments.

Virus-induced microbial mortality (*M_v_*, expressed as percent) was calculated from viral production (*P_v_*, virions cm^-3^ hr^-1^), burst size (*B*, virions cell^-1^) and microbial growth rate (*μ*, cells cm^-3^ hr^-1^), as *M_v_* = *P_v_* ⋅*B*^-1^ ⋅*μ*^-1^ ⋅ 100. *M_v_* ranged from 8 to 29%, with greater values in LH compared to GH4 (**Table 2**). Uncertainties in measured abundances of both microbes and viruses were propagated into these calculations (Supplementary Table S4). With notable exceptions (Hewson and Fuhrman, 2003; Mei and Danovaro, 2004) these values are lower than are typically reported from marine sediments. The highest *M_v_*s (up to 100%) were found in coastal sediments off Chile (Middelboe and Glud, 2006) and in coastal Adriatic sediments (57%; Mei and Danovaro, 2004). Danovaro et al.’s (2008b) assessment of globally distributed marine surface sediments estimated 80% *M_v_*. Similarly, studies of *M_v_* in deep-sea sediments (Middelboe et al., 2006; Corinaldesi et al., 2010) reported that viruses play a crucial role in the mortality of deep benthic microbes, and are responsible for up to 60% of microbial mortality. Prior to this work, we had hypothesized that low biomass, slow microbial growth, and infrequent viral-microbe contact rates would result in limited viral-induced microbial mortality, possibly alluding to a trophic lower limit to viral activity. However, in this system, viruses may have adapted to their hosts’ relatively slow growth rates and low densities by adopting both physical characters that resist decay, prolonging their opportunity to contact a suitable host, and by reducing their burst size, necessitating fewer of their host’s energetic resources in order to propagate. Similarly to cold-active phage 9A, there may also be selection for broad host range under the low contact frequency conditions in these sediments (Wells and Deming, 2006; Colangelo-Lillis and Deming, 2013). Viruses are unlikely to be the dominant cause of microbial mortality in these cold hypersaline oligotrophic sediments but do appear to be active and may yet have a significant impact on carbon and nutrient flow in these sediments.

### Dissolved Organic Carbon and the Viral Shunt

Viral infection has the potential to stimulate microbial growth by increasing nutrient availability through cell lysis and the liberation of soluble cytoplasmic components. This viral shunt has important ecological and biogeochemical consequences in the surface ocean (Suttle, 2007), marine surface sediments (Danovaro et al., 2008b), and potentially subsurface sediments (Engelhardt et al., 2014). Employing a microbial N/C- and P/C-ratio of 0.26 and 0.04 (Weinbauer and Höfle, 1998), lysis of cells in GH4 and LH sediments released organic carbon at rates between 0.18 and 140 × 10^-2^ ng C cm^-3^ h^-1^; organic nitrogen at rates between 0.48 and 370 × 10^-3^ ng N cm^-3^ h^-1^; and phosphorous at rates between 0.74 and 560 × 10^-4^ ng P cm^-3^ h^-1^ (Fischer et al., 2006). These rates are one to three orders of magnitude lower than the lowest rates reported for surface (1 cm below sea floor) sediments (Danovaro et al., 2008b), and thus are not expected to stimulate microbial growth to a degree comparable to those sediments. Extending these findings, the sediments investigated here more closely resemble the bulk volume of deep subsurface marine sediments in organic carbon content, biomass, temperature, and oxygenation than do many of the other sediments yet investigated for viral dynamics, and the dynamic values here may serve as proxies for similar cold and/or hypersaline environments where these measurements have not been made (Colangelo-Lillis et al., 2016a).

### Large Fraction of Lysogens from Coldest, most Hypersaline Sediments

Lysogenic replication is more frequent in conditions unfavorable to microbial growth, while lytic replication dominates in highly productive environments (Wilcox and Fuhrman, 1994; Weinbauer and Suttle, 1996; Tapper and Hicks, 1998; Paul, 2008). Concordantly, Danovaro et al. (2008a) proposed that lysogeny might be less important than the lytic cycle in surface sediments, as those sediments generally provide abundant resources for the growth of heterotrophic microbes. However, the environmental frequency of lysogens varies widely (3–52%; Wilcox and Fuhrman, 1994; Weinbauer and Suttle, 1996; Cochran and Paul, 1998; Cochran et al., 1998; Wommack and Colwell, 2000; Paul, 2008), and recently the stringency of this trophic condition-replication strategy relationship has been called into question (Knowles et al., 2016). In surface sediments, low frequencies of lysogens have been reported, but those frequencies increase (up to five-fold) as nutrients decrease with depth (Mei and Danovaro, 2004). A hypothesis of this investigation was that lysogenic replication would be favored as a replication strategy in cold hypersaline oligotrophic sediments with low microbial growth, possibly to a greater extent than yet reported, reflecting the extremely low biomass and viral-host encounter rates (Colangelo-Lillis et al., 2016b). Inducible lysogens represented substantial fractions of the microbial communities of each spring station investigated here (15–71%; **Table 2**); these notably exceed those found in other sedimentary environments (Glud and Middelboe, 2004; Danovaro et al., 2008b). The greatest fraction of lysogens was found in the outlet sediments of LH, the spring station characterized by the lowest microbial and viral abundances, lowest microbial growth and viral production rates, and relatively low concentrations of total and organic carbon. This may have implications for microbial evolution in these sediments as proviruses can enhance host fitness (Edlin et al., 1975), and hyperhalophilic viruses display a greater rate of gene transfer and recombination (Roux et al., 2016). Compared to the decay rates of the *in situ* viral communities, induced temperate viruses decayed six-fold more slowly in GH4 channel and three-fold more quickly in LH channel. The discrepancies between these rates of decay suggest that the *in situ* labile viral populations are not the result of lysis by the substantial population of inducible temperate viruses.

## Conclusion

This work was undertaken to evaluate the role of viruses in sediment environments at the boundaries of biological and geochemical parameters thus far examined, and to test the hypothesis that a combination of cold and subzero temperatures, low nutrient concentration, low biomass and high salinity would inhibit viral replication by lysis and promote replication by lysogenic replication. Our findings indicate that these low energy sediments maintain extremely low rates of microbial growth and viral production and that a substantial fraction of viruses are extremely resistant to decay. These viruses do contribute to microbial mortality, but are not the primary cause of such, leaving the questions of mechanism of control on growth and ultimate cause of mortality open. The relatively low rates of viral-induced mortality released amounts of dissolved nutrients into the sediments insufficient to stimulate microbial growth. A substantial fraction of microbes in these sediments appear to be lysogens, harboring inducible provirus yet based on their distinct decay rates these temperate viruses do not seem to make up a substantial fraction of the *in situ* population of virions. The similarity in physical character, aqueous chemistry and abundances of microbes and viruses between these cold, oligotrophic, hypersaline sediments and subsurface marine sediments indicate that viruses may have a substantially tempered role in influencing microbial ecology in the deep subsurface sediments of the global ocean, and that these interactions are operating on substantially greater time scales.

## Author Contributions

JC-L and LW designed the study; JC-L and IR-B conducted the experiments; JC-L, BW, and LW wrote the paper.

## Conflict of Interest Statement

The authors declare that the research was conducted in the absence of any commercial or financial relationships that could be construed as a potential conflict of interest.
