# Supplementary material for: Viral Induced Microbial Mortality in Arctic Hypersaline Spring Sediments
Source: Front Microbiol. 2017 Jan 23;7:2158. doi: 10.3389/fmicb.2016.02158 (PMC5253365; doi:10.3389/fmicb.2016.02158)
Supplement: Supplementary file 1 [file Data_Sheet_1.pdf]

Supplementary Material

Viral induced mortality in Arctic hypersaline spring sediments

Authors

Jesse Colangelo-Lillis[1,2*], Boswell A. Wing[1,2], Isabelle Raymond-Bouchard[3], Lyle G. Whyte[2,3]

Affiliations

[1]McGill University, Department of Earth and Planetary Science, Montreal, Quebec, Canada

[2]McGill Space Institute, McGill University, Montreal, Quebec, Canada

[3]McGill University, Department of Natural Resource Science, Montreal, Quebec, Canada

[*]**Correspondence**

Jesse Colangelo-Lillis**,** McGill University, Department of Earth and Planetary Science, Adams Building, 3450 University Street, Montreal, Quebec H3A OE8

Tel: 514.398.6767 | Fax: 514.398.4680

jesse.colangelo-lillis@mail.mcgill.ca

1 Supplementary Figures and Tables

1.1 Supplementary Figures

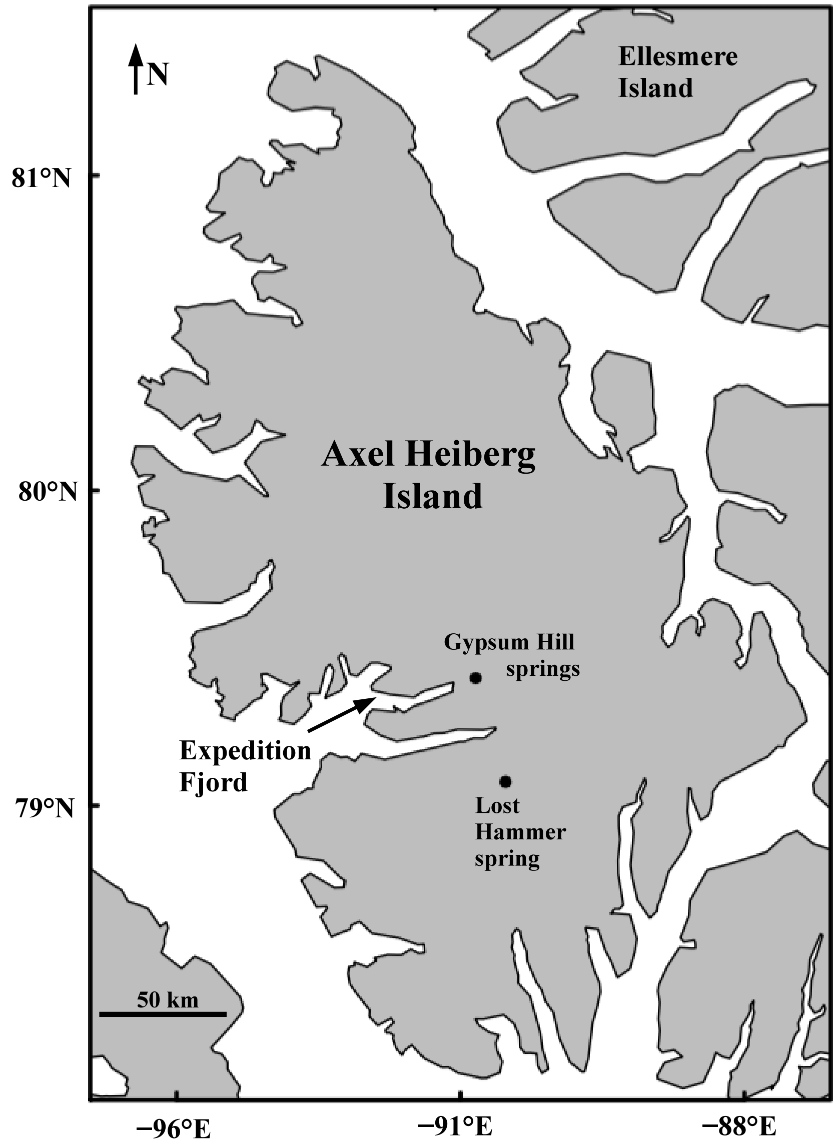

Supplementary Figure S1. Locations of hypersaline springs investigated for viral dynamics.

1.2 Supplementary Tables

Supplementary Table S1. Historical physical and spring and pore water chemistry measurements from Gypsum Hill and Lost Hammer springs.

Spring	Station	Study 1st Author. Year	year.season*	substrate	temperature (°C)	salinity (‰)	pH	ORP (mV)	dissolved oxygen (μM)	organic carbon (%)	dissolved organic carbon (mM)	sulfate (mM)	sulfide (μM)	nitrate (μM)	nitrite (μM)	ammonia (μM)	CO_2 (μM)
**Gypsum Hill**	**Outlet**	Pollard. 1999	1993-1998	water	6.0	138	8.4	na	na	na	na	39, 41	0.8	na	na	na	na
'	'	Anderson. 2004	nr, < 2004	water	6.6	100	7.7	−97.1	na	na	bdl	39	na	bdl	bdl	na	na
'	'	Perreault. 2007	2004/5.summer	water	6.9	75	7.4	−287	6.3	na	na	na	na	810	na	na	na
'	'	Colangelo-Lillis. 2016	2013.summer	water	4.2	82	7.4	−318	2.5	na	na	36	0.8	na	na	na	na
'	'	'	'	sediment	4.2	82	7	−287	na	na	na	na	na	na	na	na	na
'	'	this report	2015. summer	water	7.0	na	6.5	na	2.8, 6.6	na	0.2, 0.8	33	151	15	0.14	3.3	329
'	'	'	'	sediment	7.0	na	na	na	0, 1.3	0.15, 0.18	na	41	53	na	0.29	10.6	na
'	**Channel**	Perreault. 2007	2004/5.summer	sediment	na	na	na	na	na	0.37	na	20	na	na	na	na	na
'	'	Niederberger. 2009	2007.spring	water	5.0, 6.9	na	na	na	31	na	na	na	625	na	na	na	na
'	'	Colangelo-Lillis. 2016	2013.summer	water	na	na	na	na	na	na	na	na	na	na	na	na	na
'	'	'	'	sediment	7.8	na	na	na	na	na	na	na	na	na	na	na	na
'	'	this report	2015. summer	water	7.5	na	6.0, 6.5	na	31, 228	na	0.3, 0.5	21	299	na	2.46	3	< 227
'	'	'	'	sediment	7.5, 8.7	na	7.7	−330	0, 109	0.42, 0.45	na	39	179	26	0.43	0.3	343
**Lost Hammer**	**Outlet**	Niederberger. 2010	2005-2008	water	−5.9, −4.7	220, 260	6.0, 7.4	−187, 154	3.1, 31	na	na	54	0, 1560	na	na	na	na
'	'	'	'	sediment	na	na	na	na	na	0.45	na	1000	na	bdl	bdl	na	na
'	'	Lay. 2012	2008-2010	water	na	na	na	na	na	na	na	na	na	0, 2.6	0, 2.4	504	na
'	'	'	'	sediment	na	na	na	na	na	na	na	na	na	0, 58	0, 52	187	na
'	'	Lamarche-Gagnon. 2015	2011-2012	water	−7.7, −3.1	220, 260	6.0, 6.8	−224, −165	1.3, 35	na	na	na	0.5, 18	na	na	na	na
'	'	Colangelo-Lillis. 2016	2013.summer	water	−4.8	225	6.8	−181	0.6	na	na	na	na	na	na	na	na
'	'	this report	2015. summer	water	−4.8	na	6.1	−391	5.0	na	na	na	na	na	na	na	852
'	'	'	'	sediment	na	na	na	na	na	0.33	na	na	na	na	na	na	na
'	**Channel**	Lay. 2012	2008-2010	water	−18, −9.2	220, 260	6.5, 7.3	na	> 31	na	na	na	0, 625	0, 3.42	0, 3.42	46, 482	na
'	'	'	'	sediment	−18, 0	na	na	−30, 126	na	0.77, 0.93	na	na	na	0, 2.42	0, 2.17	56, 65	na
'	'	this report	2015. summer	water	−1.1	na	na	na	313	na	na	46, 156	4, 12	3	0.14, 0.32	300	1988
'	'	'	'	sediment	0.3, 1.6	na	na	na	35, 58	0.46, 0.54	na	75	19	na	na	na	na

*Where no season indicated, report covers multiple seasons. See reference for further detail.
For each parameter, minimum and maximum values are separated by a comma
bdl- Below detection limits.
Single quotation ' indicates value equivalent to cell immediately above.

References

Andersen, D.T. (2004) Perennial springs in the Canadian High Arctic: Analogs of Martian hydrothermal systems. *PhD Diss. McGill Univ.*

Colangelo-Lillis, J., Wing, B.A., and Whyte, L.G. (2016) Low viral predation pressure in cold hypersaline Arctic sediments and limits on lytic replication. *Environ. Microbiol. Rep.*

Lamarche-Gagnon, G., Comery, R., Greer, C.W., and Whyte, L.G. (2015) Evidence of in situ microbial activity and sulphidogenesis in perennially sub-0 °C and hypersaline sediments of a high Arctic permafrost spring. *Extremophiles* **19**: 1–15.

Lay, C.Y., Mykytczuk, N.C.S., Niederberger, T.D., Martineau, C., Greer, C.W., and Whyte, L.G. (2012) Microbial diversity and activity in hypersaline high Arctic spring channels. *Extremophiles* **16**: 177–191.

Niederberger, T.D., Perreault, N.N., Lawrence, J.R., Nadeau, J.L., Mielke, R.E., Greer, C.W., et al. (2009) Novel sulfur-oxidizing streamers thriving in perennial cold saline springs of the Canadian high Arctic. *Environ. Microbiol.* **11**: 616–629.

Niederberger, T.D., Perreault, N.N., Tille, S., Lollar, B.S., Lacrampe-Couloume, G., Andersen, D., et al. (2010) Microbial characterization of a subzero, hypersaline methane seep in the Canadian High Arctic. *ISME J.* **4**: 1326–1339.

Omelon, C.R., Pollard, W.H., and Andersen, D.T. (2006) A geochemical evaluation of perennial spring activity and associated mineral precipitates at Expedition Fjord, Axel Heiberg Island, Canadian High Arctic. *Appl. Geochemistry* **21**: 1–15.

Perreault, N.N., Andersen, D.T., Pollard, W.H., Greer, C.W., and Whyte, L.G. (2007) Characterization of the prokaryotic diversity in cold saline perennial springs of the Canadian high arctic. *Appl. Environ. Microbiol.* **73**: 1532–1543.

Pollard, W., Omelon, C., Andersen, D., and McKay, C. (1999) Perennial spring occurrence in the Expedition Fiord area of western Axel Heiberg Island, Canadian High Arctic. *Can. J. Earth Sci.* **36**: 105–120.

Supplementary Table S2. Growth rate calculations.

Spring	Station	Growth evaluated by EdU-Alexafluor (# new cells cm^{-3} h^{-1})			Growth evaluated by total cell counts (# new cells cm^{-3} h^{-1})			Corrected growth evaluated by total cell counts* (# new cells cm^{-3} h^{-1})			EdU incorporation efficiency (%)		In situ growth (# new cells cm^{-3} h^{-1})	C-appended growth rate factor	
		EdU	EdU+L	EdU+A	EdU	EdU+L	EdU+A	EdU	EdU+L	EdU+A	EdU+L	EdU+A		L	A
GH4	Outlet	4190	8320	7140	22900	74900	150000	0	52000	127000	6.3	17.8	5.04E+04	1.04	2.53
GH4	Channel	34900	71300	59600	293000	731000	1400000	0	438000	1100000	6.1	18.5	4.30E+05	1.02	2.57
LH	Outlet	56	112	96	117	1600	676	0	1480	559	13.3	5.8	5.36E+02	2.77	1.04
LH	Channel	900	633	1020	−4136	12700	16300	0	16800	20500	26.5	2.0	2.10E+04	0.80	0.97

*values corrected to 0 growth in microcosms unappended with carbon, and in dynamic equilibrium, this correction is based on the presumption that EdU can serve as a nutrient (personal correspondence with Invitrogen technical representative)

Supplementary Table S3. Provirus induction calculations.

Spring	Station	Induction period (h)	Microbial reduction (cells cm^{-3})	Microbial reduction (%)	Induced viral production (VLP cm^{-3})	Burst size (VLP $cell^{-1}$)	Induced temperate viral decay rate (VLP cm^{-3} h^{-1})	Endogenous viral decay rate (VLP cm^{-3} h^{-1})	Induced/ Endogenous decay ratio
GH	Outlet	24	1.2×10^{6}	15	2.7×10^{7}	21	na	8.3×10^{4}	na
GH	Channel	24	2.6×10^{7}	34	5.6×10^{8}	22	2.9×10^{5}	1.7×10^{6}	0.17
LH	Outlet	144	2.4×10^{5}	71	2.2×10^{6}	9	na	na	na
LH	Channel	48	1.3×10^{6}	42	1.2×10^{7}	12	1.4×10^{5}	7.4×10^{4}	1.95

Supplementary Table S4. Uncertainties of microbial and viral abundances and rate calculations. Uncertainties of abundances are reported as the standard deviation between biological replicates of untreated time 0 h and 96 h, and treated 0 h samples. Uncertainties for calculated values are propagated from uncertainties on abundance measurements by adding the fractionation uncertainty in each component of the calculation for products.

Spring. Station	Microbial growth (cm^{-3} h^{-1})	Viral production (cm^{-3} h^{-1})	Burst size (virions $cell^{-1}$)	Inducible microbes (%)	Microbial mortality (%)
GH.Outlet	$5.0 \pm 0.4 \times 10^{4}$	$8.3 \pm 1.6 \times 10^{4}$	21±6	15.0±1.3	7.9±4.4
GH.Channel	$4.3 \pm 0.2 \times 10^{5}$	$1.7 \pm 0.3 \times 10^{6}$	22±4	33.8±1.3	18.4±6.8
LH.Outlet	$5.4 \pm 1.2 \times 10^{2}$	$9.1 \pm 2.6 \times 10^{2}$	9±4	70.6±16.0	18.9±16.5
LH.Channel	$2.1 \pm 0.5 \times 10^{4}$	$7.4 \pm 1.5 \times 10^{4}$	12±5	43.3±10.5	29.4±26.6
